# Chromatin-Based Regulation of Plant Root Development

**DOI:** 10.3389/fpls.2018.01509

**Published:** 2018-10-16

**Authors:** Dong-Hong Chen, Yong Huang, Changhua Jiang, Jin-Ping Si

**Affiliations:** ^1^State Key Laboratory of Subtropical Silviculture, SFGA Engineering Research Center for Dendrobium Catenatum, Zhejiang A&F University, Hangzhou, China; ^2^Key Laboratory of Education Department of Hunan Province on Plant Genetics and Molecular Biology, Hunan Agricultural University, Changsha, China; ^3^Shanghai Botanical Garden, Shanghai, China

**Keywords:** chromatin structure, histone modification, Polycomb, TrxG, SDG family, chromatin remodeling, root development, stem cell niche

## Abstract

Plant is endowed with sessile habit and nutrient acquisition mainly through the root organ, which also provides an excellent model to study stem cell fate and asymmetric division due to well-organized cell layers and relatively simple cell types in root meristem. Besides genetic material DNA wrapped around histone octamer, chromatin structure determined by chromatin modification including DNA methylation, histone modification and chromatin remodeling also contributes greatly to the regulation of gene expression. In this review, we summarize the current progresses on the molecular mechanisms of chromatin modification in regulating root development.

## Introduction

The root system constitutes the underground world of a plant, and it takes charge of nutrient and water supply for the whole plant under normal and stress environments. The developmental status of the root system directly determines the plant survival and performance of leaves, stems, flowers, fruits, and seeds, which are closely related to animal and human life. Compared with the aerial part, the building unit of a root system is simple and mainly consists of primary and lateral roots (LRs) without substantial difference in appearance. An intact root system is derived from the tiny root apical meristem (RAM). Proliferation and differentiation of RAM give rise to different types of root cells and tissues.

Transcription factors combined with phytohormones, small signaling molecules, and miRNAs play an essential role in regulating the stem cell fate and RAM maintenance during root development (Drisch and Stahl, 2015). Root development-controlled genes are stored in the genomes of all cells from the same plant, but their transcripts are only expressed in specific root cells and tissues. This kind of spatiotemporal divergence of gene expression is closely associated with the alteration in chromatin structure, which determines the transcriptional accessibility to DNA in response to internal and external stimuli (Narlikar et al., 2002). The accessible (active/open) or inaccessible (repressive/close) state of chromatin structure is mainly accomplished by three types of chromatin-based regulation, namely, DNA methylation, ATP-dependent chromatin remodeling and histone covalent modification.

## Root organogenesis in arabidopsis

Roots have two fundamental functions: anchorage to the substrate and uptake of water and nutrients in almost all plants. However, in specific species, root have evolved some novel roles, such as physical support, photoassimilate storage, metabolite biosynthesis, and interactions with the rhizosphere (Zhu et al., 2011). Roots are divided into primary roots, LRs, and adventitious roots based on position origin. Primary root initiation in the model plant Arabidopsis can be traced back to embryogenesis. Hypophysis, the uppermost suspensor cell incorporates into the embryo proper at an early globular stage. This cell divides asymmetrically, generating an upper lens-shaped quiescent center (QC) cell and a lower cell (columella initial), and finally giving rise to RAM (De Smet et al., 2008). RAM is located at the primary root tip, and it is responsible for the establishment of a post-embryonic root system. In Arabidopsis, RAM undergoes asymmetrical cell division to give rise to self-renewal and production of various cell types, which further expand and differentiate to form elongation and maturation/root hair zones. RAM is composed of the proliferation domain (PD) with high mitotic activity and the transition domain (TD) with low mitotic activity along the longitudinal axis (Ivanov and Dubrovsky, 2013). In the PD region, the stem cell niche (SCN) is located at the center, which consists of a small group of mitotically less active quiescent center (QC) cells surrounded with four types of stem cells, namely, columella initial/stem cell (CSC), epidermal cell and lateral root cap (Epi/LRC) initial, cortex/endodermis initial (CEI), and stele initial (Scheres et al., 1994). At the proximal part, CEIs divide periclinally, producing two types of ground tissues, namely, cortex and endodermis. Stele initials produce a vascular cylinder. At the distal part, Epi/LRC initials sequentially divide anticlinally, and periclinally divisions, thereby generating epidermis (Epi) and LRC. Asymmetric divisions of CSCs experience directly generate differentiated columella cells with starch granules for gravity sensing (Dolan et al., 1993). LR branching from the parent root is crucial for maximizing the function of the root system. In angiosperms, LRs are usually derived from the pericycle cells of parent roots. However, endodermis cells contribute to LR formation in some monocots (Malamy and Benfey, 1997). In Arabidopsis, the organogenesis of a LR can be divided into eight stages based on specific anatomical characteristics and cell divisions (Malamy and Benfey, 1997). In summary, LRs are initiated when single or pairs of pericycle founder cells across the xylem poles undergo several rounds of asymmetric anticlinal divisions, creating a single layered primordium containing up to 10 small cells (stage I). Such cells divide periclinally and form an inner layer and outer layer (stage II). After a series of anticlinal and periclinal divisions, the LR primordium (LRP) gradually acquires a dome shape (stages III to VII) and eventually protrudes from the parent root (stage VIII) (Malamy and Benfey, 1997; Casimiro et al., 2001; Dubrovsky et al., 2001; Peret et al., 2009). Adventitious roots indicate shoot-born roots from undifferentiated and reprogrammed cells, which are often associated with vascular tissues in response to developmental and environmental stimuli. adventitious root emergence shares many developmental characteristics with LR formation, but it requires a specific set of signaling components (Verstraeten et al., 2014). Additionally, root hairs in soil strengthen the absorption capability of roots. A root hair is derived from a specific root epidermal cell, and its development has four phases: cell fate specification of trichoblasts and atrichoblasts, root hair initiation, tip growth and maturation (Gilroy and Jones, 2000).

## Chromatin structure and chromatin modifiers

Nucleosome, the fundamental building unit of chromatin, is composed of ~146 bp of DNA wrapped around a histone octamer with two copies of each of the core histones H2A, H2B, H3, and H4 (Luger et al., 1997). Nucleosomes are interconnected by linker DNAs and fixed by histones H1, which are involved in higher-order chromatin structure compaction. The status of DNA packaging into chromatin affects the accessibility of DNA by regulatory factors and the output of genomic information at specific spatio-temporal developmental contexts. The chromatin structure can be regulated by chromatin remodelers, which disrupt non-covalent DNA-histone interactions and histone modifiers which catalyze or remove covalent linkages of chemical groups from histones (Li et al., 2007). ATP-dependent chromatin remodeling enzymes alter the chromatin structure by adjusting the density or location of nucleosomes on the target DNA locus and catalyze the incorporation of histone variants using ATP hydrolysis energy (Narlikar et al., 2013). On the basis of phylogenetic analysis, chromatin remodelers in plants can be divided into 18 classes, and each class harbors a common catalytic ATPase domain and class-specific domains, such as well-characterized PHD, chromodomain, bromodomain, SANT, and RING domain (Knizewski et al., 2008; Hu et al., 2013). By contrast, both freely stretched tails and core regions in the histone octamer may undergo various post-translational modifications, such as methylation, acetylation, phosphorylation, ubiquitination, citrullination, hydroxylation, O-GlcNAcylation, and ADP-ribosylation (Huang et al., 2014; Lawrence et al., 2016). The dynamic balances of distinct histone modifications *in vivo* are maintained by specific enzymes (“writers” and “erasers”). For instance, active marker histone acetylation and repressive marker histone deacetylation are catalyzed by histone acetyltransferases (HATs) and histone deacetylases (HDACs), respectively (Eberharter and Becker, 2002). Histone modification either directly influences the chromatin structure by altering the charge of histone or acts as an anchorage site that is recognized by “readers,” which recruit other effectors to regulate gene expression. Specific histone modifications play fixed roles in transcription. For example, H3K4 trimethylation (H3K4me3) and H3K36me3 are associated with transcriptional activation, whereas the trimethylation of lysine 27 on histone H3 (H3K27me3) and H3K9me2 are associated with transcriptional repression (Pontvianne et al., 2010).

Chromatin modifiers normally act as part of large multimeric complexes to exert precise biological functions. These modifiers are often classified into two groups with antagonistic roles, the polycomb group (PcG) proteins associated with repressive genes and the trithorax group (TrxG) proteins associated with active genes (Schuettengruber and Cavalli, 2009; Kohler and Hennig, 2010). In animals and plants, PcG proteins mainly constitute two evolutionarily conserved complexes, namely, polycomb repressive complex 1 (PRC1) and PRC2. On the basis of the classical model in animals, PRC2 complex catalyzes H3K27me3 at the target gene chromatin, and PRC1 recognizes this repressive mark and catalyzes monoubiquitination of lysine 119 on histone H2A (H2Aub1) (Schuettengruber et al., 2007). In Arabidopsis, PRC2 core subunits contain three *Drosophila E*(*z*) homologs (CLF, SWN, and MEA), three Su(z)12 homologs (EMF2, FIS2 and VRN2), a single ESC homolog (FIE), and five p55 homologs (MSI1–MSI5). PRC1 core subunits consist of two RING1 homologs (AtRING1a/b), three Psc homologs (AtBMI1a/b/c), a Pc equivalent (LHP1), and a plant-specific component EMF1, without the Ph counterpart (Chen D. H. et al., 2016; Forderer et al., 2016).

In addition, DNA methylation is another kind of chromatin-based modification that occurs on the DNA-specific cytosine residues, such as CG, CHG, and CHH contexts (where H = A, T, or C; Law and Jacobsen, 2010). DNA methylation is widely involved in different biological programs and responses to environmental stimuli (Jullien et al., 2012; Zhang et al., 2018). However, investigation on the function of DNA methylation in root development are limited. A comparison of distinct cell types in RAM reveals the highest level of DNA methylation exists in columella root cap (Kawakatsu et al., 2016). Thus, this review emphasizes the role and regulation of histone modifiers and chromatin remodeling ATPases in root development, especially in Arabidopsis (Table 1).

**Table 1:** Chromatin modifiers in Arabidopsis.

**Mutant**	**Module or complex**	**Direct targets**	**Histone mark change**	**Root phenotype**	**References**
**HISTONE ACETYLATION**					
*gcn5/hag1*	GCN5-ADA2b, GANT family	[PLT]	n.d.	Shorter root; premature RAM	Kornet and Scheres, 2009
		n.d.	n.d.	Altered epidermal cell patterning	Chen W. Q. et al., 2016
*haf2*	TAFII250 family	n.d.	n.d.	Altered epidermal cell patterning	Chen W. Q. et al., 2016
*hdt1 hdt2*	HDT family	GA2ox2	Reduced H3ac	Reduced RAM cell number	Li H. et al., 2017
**HISTONE DEACETYLATION**					
*hda18*	Class-II HDAC	Four kinase genes	increased H3ac or H4ac	Altered epidermal cell patterning	Xu et al., 2005; Liu et al., 2013
*hda6*	Class-I HDAC	ETC1, GL2	increased H3ac and H4ac	Altered epidermal cell patterning	Li et al., 2015
*hda19*	Class-I HDAC	n.d.	n.d.	Altered epidermal cell patterning	Chen W. Q. et al., 2016
*ldl1/swp1*	LDL1-HDAC corepressor	LRP1	Increased H3ac and H4ac	Longer root	Krichevsky et al., 2009
**HISTONE METHYLATION-RELATED**					
*clf*	PRC2	AGL42/17/21, PLT1/2, WOX5	Reduced H3K27me3	Larger RAM, longer root	Aichinger et al., 2011
		PIN1	n.d.	Increased LR number	Gu et al., 2014
*emf2*	PRC2	n.d.	n.d.	Longer root, increased LR number	Gu et al., 2014
*swn*	PRC2	n.d.	n.d.	Shorter root	de Lucas et al., 2016
*clf swn*	PRC2	WIND3, LEC2	Loss of H3K27me3	Somatic embryo from single root hair, pkl-type root	Ikeuchi et al., 2015
*fie*	PRC2	WIND3, LEC2	Loss of H3K27me3	Somatic embryo from single root hair, pkl-type root	Bouyer et al., 2011; Ikeuchi et al., 2015
		n.d.	n.d.	Reduced RAM, larger stele (Knockdown)	de Lucas et al., 2016
*emf2 vrn2*	PRC2	WIND3, LEC2	Loss of H3K27me3	Somatic embryo from single root hair; pkl-type root	Ikeuchi et al., 2015
*msi1*	PRC2	n.d.	n.d.	Shorter root	de Lucas et al., 2016
*sdg2*	TrxG, H3K4me3 writer	n.d.	Reduced H3K4me3	Shorter root, reduced LR number	Yao et al., 2013
*atx1*	TrxG, H3K4me3 writer	n.d.	n.d.	Shorter root	Napsucialy-Mendivil et al., 2014
*ashr3*	H3K36me1 writer	n.d.	reduced H3K36me1	Shorter root	Kumpf et al., 2014
**HISTONE MONOUBIQUITINATION**					
*atring1a 1b*	PRC1, H2Aub1 writer	[Seed maturation genes]	n.d.	pkl-type root	Chen et al., 2010
*atbmi1a 1b* (*1c*)	PRC1, H2Aub1 writer	LEC1, FUS3, ABI3, BBM	Loss of H2Aub1, reduced H3K27me3	pkl-type root	Yang et al., 2013
**HISTONE MODIFICATION READER**					
*lhp1*	PcG, H3K27me3 reader	MGP	n.d.	Premature middle cortex formation	Cui and Benfey, 2009
*val1 val2*	H3K4me3/H3K27me3 reader	LEC1, FUS3, ABI3, BBM	Reduced H2Aub1 and H3K27me3	pkl-type root	Yang et al., 2013; Chen et al., 2018
*al6*	H3K4me3 reader	[ETC1, NPC4, SQD2, PS2]	n.d.	Very short root hair under Pi starvation	Chandrika et al., 2013a,b
*row1*	H3K4me3 reader	WOX5	n.d.	Extremely short root, loss of gravitropic response	Zhang et al., 2015
**CHROMATIN REMODELING**					
*pkl/ssl2*	CHD3 family, TrxG	AGL42/17/21, PLT1/2, WOX5	Increased H3K27me3	Shorter root	Aichinger et al., 2011
*pkl* (*pkr2*)	CHD3 faimly, TrxG	EMF2, SWN	Reduced H3K27me3	pkl-type root	Aichinger et al., 2009
*brm*	SWI/SNF family, TrxG	PIN1~4, PIN7	Reduced H3K27me3	Shorter root, decreased RAM size, increased LR number	Yang et al., 2015
*baf60*	SWI/SNF complex subunit	IPT3, IPT7, KRP7	Decreased H3K27me3, increased H3K4me3	Short root, reduced LR number	Jegu et al., 2015
*chr12 chr23*	SWI/SNF family	WOX5	n.d.	No RAM initiation (strong), much short root (weak)	Sang et al., 2012

*[]Putative targets lacking evidence of direct binding. n.d., not detected*.

## Histone acetylation and deacetylation in root development

The homeostasis of histone acetylation *in vivo* is dynamically maintained by HATs and HDACs (Shahbazian and Grunstein, 2007). Arabidopsis possesses 12 HATs and 18 HDACs, in which HATs are categorized into four families (GNAT/HAG, MYST/HAM, p300/CBP/HAC, and TAFII250/HAF), and HDACs are composed of three classes (RPD3, HDA1, and SIR2) homologous to yeast counterparts, and a plant-specific class (HD2; Pandey et al., 2002; Hollender and Liu, 2008).

Histone acetylation plays a key role in regulating the cellular patterning of the root epidermis. The single-layered epidermis in Arabidopsis root harbors two cell types, namely, trichoblast/hair cell (H) and atrichoblast/non-hair cell (N) in an alternative arrangement, providing a well-characterized system for studying the relationship between root epidermal cell fate decision and positional cue from underlying cortical cells (Caro et al., 2007). Treatment with HDAC inhibitor Trichostatin A (TSA) induces H development at N positions in the root epidermis through hyperacetylation of histones H3 and H4 and ectopic expression of patterning genes *CPC, GL2*, and *WER* (Xu et al., 2005). However, the mutation and overexpression of histone deacetylase *HDA18* result in the conversion of cells at the N position to H fate; HDA18 is required for the establishment of root epidermal cell patterning through regulating histone acetylation levels of several kinase loci but not pattern genes (Xu et al., 2005; Liu et al., 2013). Similarly, *HDA6* depletion leads to ectopic H cells at the N positions; HDA6 affects cellular patterning by altering the status of histone acetylation at the promoter regions of pattern genes *ETC1* and *GL2* (Li et al., 2015). Phenotypic analysis of all available HDAC and HAT single mutants further shows three HDACs (HDA6, HDA18, and HDA19) and two HATs (GCN5 and HAF2) exhibit altering cellular patterns in root epidermis (Chen W. Q. et al., 2016).

Histone acetylation is important for root growth and morphogenesis. HDAC inhibitors repress primary root elongation and LR emergence via regulation of 26S proteasome-mediated degradation of PIN1 and alteration in auxin distribution in Arabidopsis (Nguyen et al., 2013). Furthermore, TSA treatment affects the expression of S-phase kinase-associated protein 2B (SKP2B), which acts as an F-box protein involved in the regulation of the stability of cyclin-dependent kinase inhibitor KRP1 (Ren et al., 2008). *SKP2B* promoter is regulated by histone H3 acetylation on K9 and K14 in an auxin- and IAA14/SLR-dependent manner, and *skp2b* mutant displays increased root elongation and LR emergence (Manzano et al., 2012). On the other hand, TSA can rescue LR formation in the gain-of-function mutant *slr-1*, which has stabilized mutant SOLITARY-ROOT (SLR)/IAA14 (mIAA14) protein with enhanced inhibiting capacity on the downstream targets *ARF7*/*19* (Fukaki et al., 2006). In rice, overexpression of a class I-type (RPD3-like) histone deacetylase *OsHDAC1* accelerates root growth rate in seedlings (Jang et al., 2003). Chromatin immunoprecipitation (ChIP) assay showed that OsHDAC1 represses the key downstream target *OsNAC6* by binding to the *OsNAC6* promoter and deacetylating K9, K14, and K18 on H3 and K5, K12, and K16 on H4. Consistently, *OsNAC6* knockout seedlings have a root phenotype similar to *OsHDAC1* overexpression seedlings (Chung et al., 2009). In *Populus*, HDACs are required for *de novo* organogenesis and normal development of roots, and TSA treatment delays the regeneration of adventitious roots and inhibits the growth of primary roots, similar to Arabidopsis (Ma et al., 2016). *LDL1/SWP1* encodes a homolog of human *LYSINE-SPECIFIC DEMETHYLASE1* (*LSD1*), which is a core component of several HDAC corepressor complexes (Humphrey et al., 2001; Hakimi et al., 2002; Jiang et al., 2007). *LDL1/SWP1* participates in regulating root elongation by suppressing the root primordium-specific gene *LRP1* via histone deacetylation. Similar to the phenotype of *LRP1* overexpression, Loss of function of *LDL1* leads to histone hyperacetylation and elevated expression of *LRP1*, thereby increasing root elongation (Krichevsky et al., 2009).

Histone acetylation is required for RAM maintenance and cell proliferation and differentiation. TSA treatment causes aberrant SCN cell divisions and formation of an extra layer of ground tissue (premature middle cortex) in roots (Cui and Benfey, 2009). Histone acetyltransferase GENERAL CONTROL NON-DEREPRESSIBLE 5 (GCN5) and the associated factor ALTERATION/DEFICIENCY IN ACTIVATION 2b (ADA2b) act in the PLT pathway and mediate the proliferation of transit-amplifying cells during root development (Vlachonasios et al., 2003; Kornet and Scheres, 2009). However, GCN5, but not ADA2b, is involved in root SCN maintenance, and *PLT2* overexpression restores the SCN defect of *gcn5* mutants. Therefore, ADA2b and GCN5 have both similar and distinct functions. HDT1/2 determine the number of RAM cells by influencing the transition from cell division to expansion. Downregulation of *HDT1/2* in *hdt1,2i* causes earlier transition from cell division to expansion, reduced the number of RAM cells. Furthermore, HDT1/2 repress the expression of *GA2ox2* in the RAM and elongation zone through histone deacetylation of *GA2ox2*, thereby increasing the expression of *hdt1,2i* plants. Moreover, *GA2ox2* overexpression in RAM phenocopies *hdt1,2i* mutants, whereas *GA2ox2* knockout partially rescues *hdt1,2i* root defects (Li H. et al., 2017). WUSCHEL-related homeobox (WOX) transcription factors are the master regulators that maintain stem cell fate in different meristem types, such as shoot and root meristems, vascular cambia, and leaf marginal meristems (Aichinger et al., 2012). WOX5 is a mobile organizer signal that moves from the QC to the neighboring CSCs, and recruits Groucho co-repressors TOPLESS/TOPLESS-RELATED (TPL/TPR) and HISTONE DEACETYLASE 19 (HDA19) to induce histone deacetylation and repress the differentiation factor *CYCLING DOF FACTOR 4* (*CDF4*), resulting in formation of decreased CDF4 protein gradient from differentiated columella, CSCs to QC, which is opposite to the WOX5 protein gradient and permits the exit of stem cell descendants from the stem cell state (Pi et al., 2015). In rice, *OsWOX11* stimulate meristem cell proliferation in crown roots via recruitment of the ADA2-GCN5 histone acetyltransferase module to activate downstream target genes (Zhou et al., 2017).

Histone acetylation is involved in the regulation of root development in response to environmental stress. In maize seedlings, exposure to salt stress results in shortening and swelling of roots due to reduced meristematic activity and enlargement of stele tissues and cortex cells in the elongation zone (Li et al., 2014). NaCl treatment increases the expression of histone acetyltransferases *ZmHATB* and *ZmGCN5* and the global acetylation levels of H3K9 and H4K5; consistently, cell wall-related genes *ZmEXPB2* and *ZmXET1* are upregulated through the increased deposition of H3K9 acetylation on their promoter and coding regions (Li et al., 2014). Thus, histone acetylation is involved in the rapid regulation of the expression of cell wall-related genes, which mediate cell enlargement and reduce salinity-induced ionic toxicity.

## Histone methylation and PRC2 complex in root development

Histone methylation is mainly catalyzed by evolutionarily conserved SET domain-containing proteins of the SET-DOMAIN GROUP (SDG) family (Ng et al., 2007). The Arabidopsis genome contains 49 SDG members, which can be divided into seven classes with distinct functions, E(z) homologs for H3K27 methylation, ASH1 homologs for H3K36 methylation, TRX homologs for H3K4 methylation, Su(var) homologs, S-ET proteins with an interrupted SET domain, and RBCMT for non-histone proteins (Ng et al., 2007; Pontvianne et al., 2010). Among these classes, E(z) homologs constitute the key catalytic subunits in multiple PRC2 complexes, involved in the regulation of cell fate transition and cell identity maintenance.

SDG family and histone methylation are essential for RAM maintenance and LR formation. SDG2 is the major H3K4 methyltransferase *in vivo* (Berr et al., 2010), and it is required for SCN maintenance in primary roots and SCN establishment in LRs. Depletion of SDG2 results in drastically reduced H3K4me3 levels and loss of auxin gradient maximum in root SCN cells. Additionally, SDG2 is involved in protecting genome integrity in SCN, and the *sdg2* mutant displays elevated DNA damage (Yao et al., 2013). Another H3K4 methyltransferase Arabidopsis homolog of trithorax1 (ATX1/SDG27) is implicated in SCN maintenance and cell patterning during root development; the loss-of-function of mutant *atx1-1* reveals the retarded growth of primary roots due to reduced RAM activity, and disorganized cell patterns due to loss of coordination between cell division and proliferation (Napsucialy-Mendivil et al., 2014). ATX1 is also required for LR initiation and morphogenesis; the *atx1-1* mutant undergoes additional anticlinal divisions in stage-I LRP, asymmetric development, and delayed LR emergence (Napsucialy-Mendivil et al., 2014). The ASHR3/SDG4 that acts as an H3K36 monomethyltransferase is expressed in SCN and is required for the synchronization of replication and cell divisions in the root proximal meristematic zone (Kumpf et al., 2014). In comparison with the synchronous switch from mitotic cell cycle to endoreduplication in WT root meristems, the mutations of ASHR3 gene disrupt the coordinated pattern of DNA replication and cell division and accelerates QC division rate (Kumpf et al., 2014). *ASHR3* is the direct downstream target of E2Fa/E2Fb transcription factors which control the G1-to-S-phase transition, thereby confirming the role of ASHR3 in the regulation of cell division (Kumpf et al., 2014). In addition, PHD-containing protein REPRESSOR OF WUSCHEL1 (ROW1) is an H3K4me3 reader that is required for QC identity maintenance and SCN development by specifically recognizing the H3K4me3 mark of the *WOX5* promoter to repress its transcription in the proximal root meristem. Mutation in *wox5* can rescue the extremely short root phenotype of *row1* (Zhang et al., 2015).

CLF is the major H3K27me3 writer in plants and it is a catalytic subunit in PRC2 complex. full catalytic activity of CLF requires the integrity of PRC2 complex due to the almost entire loss of H3K27me3 mark in other PRC2 subunit mutants, such as *emf2 vrn2* and *fie* (Schubert et al., 2006; Bouyer et al., 2011; Lafos et al., 2011). PRC2 core components CLF, SWN, EMF2, VRN2, FIE, and MSI1 but not endosperm-specific MEA are implicated in root meristem development and vascular cell proliferation and specification (de Lucas et al., 2016). The *swn* mutant has reduced root elongation without affecting meristem size, whereas *clf-29* display slightly elongated primary roots and large root meristems (Aichinger et al., 2011; de Lucas et al., 2016), *clf-28 swn-7* and *fie* mutants have substantially shortened roots with small meristems and increased number of cells in the vascular cylinder (de Lucas et al., 2016). Silencing of *MSI1* specifically in the root vascular cylinder conferred by *WOLp::amiRNA_MSI1* construction leads to moderately decreased root growth and extremely small root meristems (de Lucas et al., 2016). Similar to the *clf* mutant phenotype, the knockdown of *EMF2* results in long primary roots and curly leaves (Gu et al., 2014). Additionally, CLF associated with EMF2 (EMF-PRC2 complex) is involved in the inhibition of LR formation, and the loss-of-function of either *EMF2* or *CLF* results in greatly increased LR production along the primary roots. In the complex, CLF binds to and deposits the repressive mark H3K27me3 to the chromatin of the auxin efflux carrier gene *PIN FORMED 1* (*PIN1*); it downregulates auxin maxima in roots, thereby inhibiting the establishment of founder cells during LR initiation (Gu et al., 2014). PRC2 is involved in the repression of somatic embryo production in primary roots; the PRC2 mutants (*swn clf*, *emf2 vrn2*, and *fie*) display a certain proportion of “pkl-root” phenotype (Bouyer et al., 2011). PRC2 can prevent the unscheduled reprogramming of terminally differentiated root hair cells (Ikeuchi et al., 2015). A mature WT root hair is a unicellular structure that typically undergoes endoreduplication, which is a feature of fully differentiated cells with lost ability to re-enter the mitotic cycle (Breuer et al., 2010). The root hairs in *swn clf* and *emf2 vrn2* first complete normal endoreduplication, but then they unexpectedly reactivate mitotic division to generate the multicellular structures and embryonic calli. The PRC2 target genes *WIND3* and *LEC2* contribute to reprogramming through their overexpression, mimicking the root hair defects of PRC2 mutants (Ikeuchi et al., 2015).

Specific transcriptional factors and enzymes associated with PcG proteins can regulate downstream gene expression and root development. Similar to animal GAGA-binding factors (Berger and Dubreucq, 2012), plant-specific BASIC PENTACYSTEINE/BARLEY B RECOMBINANT (BPC/BBR) proteins can bind to the GA-repeat promoter region *in vitro* (Meister et al., 2004; Wanke et al., 2011). Arabidopsis BPC proteins, including BPC1, BPC2, BPC4, and BPC6, can directly interact with the PRC2 subunit SWN, but not with CLF, VRN2, EMF2, and FIE; BPCs promote LR development though recruiting PRC2 to deposit H3K27me3 repressive mark at the *ABI4* locus (Mu et al., 2017). BPC6 can also recruit PcG protein LHP1 to polycomb-responsive DNA element-like GAGA motifs, and serve as a scaffold for the sequential attachment of PRC2 due to LHP1 interacting with PRC2 subunit VRN2 (Hecker et al., 2015). DNA topoisomerase TOPOISOMERASE1α (TOP1α) influences PcG-mediated epigenetic regulation through reducing the nucleosome density in floral meristems (Liu et al., 2014), and it regulates root meristem activity through maintaining the genome integrity of stele stem cells and the undifferentiated state of CSCs (Zhang et al., 2016).

## Histone monoubiquitination and PRC1 complex in root development

In Arabidopsis, the histone repressive mark H2Aub1 is catalyzed by the PRC1 RING-finger proteins AtRING1a/b and AtBMI1a/b/c (Bratzel et al., 2010; Li et al., 2017), which function throughout the life cycle and are implicated in regulating seed germination, vegetative maintenance, flowering time control, floral organ identity, and fertility [(Xu et al., 2005; Bratzel et al., 2010; Chen et al., 2010; Yang et al., 2013; Chen D. et al., 2016)]. PRC1 RING-finger proteins inhibit embryonic traits in primary root mainly through repressing the ectopic expression of seed maturation genes *ABSCISIC ACID INSENSITIVE3* (*ABI3*), *FUSCA3* (*FUS3*), *LEAFY COTYLEDON2* (*LEC2*), and *LEAFY COTYLEDON1* (*LEC1*) (Bratzel et al., 2010; Chen et al., 2010). AtBMI1a/b/c can interact with B3 factors VP1/ABI3-LIKE (VAL) proteins, which are also required for preventing the vegetative-to-embryonic reversion in roots (Suzuki et al., 2007; Yang et al., 2013). Loss of VAL or AtBMI1 activity disrupts the incorporation of the repressive marks H2Aub1 and H3K27me3 in seed maturation genes due to the strongly reduced levels of these two marks in *val1 val2* and *atbmi1a atbmi1b atbmi1c* mutants (Yang et al., 2013).

**Figure 1:**
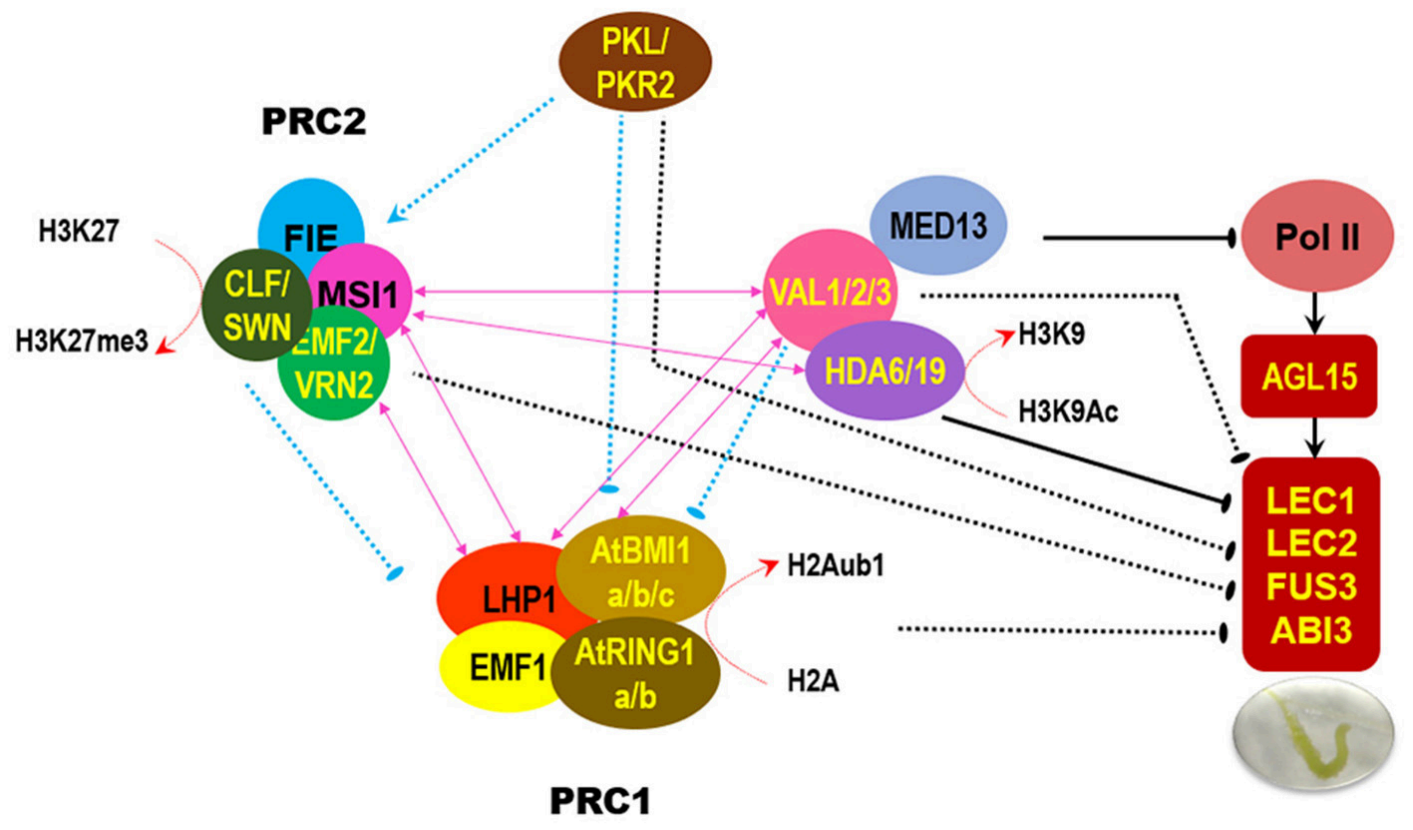
Regulatory relationship among distinct chromatin modifiers for repressing development of embryonic traits in the root. The schematic summary is mainly based on the published results (Derkacheva et al., 2013; Zhou et al., 2013; Hecker et al., 2015; Mehdi et al., 2016; Yuan et al., 2016). Pink double-headed arrow indicates direct protein-protein interaction. “T” bar indicates repressive effect, arrow indicates promotive effect; solid line means direct target gene, dash line means uncertain downstream target.

In addition to the signature B3 domain, VAL proteins harbor other conserved domains, such as the plant homeodomain (PHD) and cysteine and tryptophan residue-containing domain (CW), which usually act as histone code reader modules, and the ethylene-responsive element binding factor-associated amphiphilic repression (EAR) domain that functions as a repressive motif to recruit corepressors (Chen et al., 2018). *In vitro* assays showed that VAL1/2 may recognize dimethylated histone H3 lysine 4 (H3K4me2) and H3K4me3 through the CW domain or PHD domain and H3K27me3 through the PHD domain (Hoppmann et al., 2011; Chen et al., 2018). During the prevention of somatic embryogenesis, HIGH-LEVEL EXPRESSION OF SUGAR-INDUCIBLE GENE 2 (HSI2)/VAL1 binds to the two RY cis elements upstream of the *AGL15* gene, recruits PRC2 subunit MSI1, and promotes the deposition of repressive mark H3K27me3, thereby suppressing the seed maturation regulatory program (Chen et al., 2018). Additionally, HSI2/VAL1 can interact with HDA6 and the TRAP240 domain of MED13, which is a subunit of the conserved MEDIATOR (MED) complex linking transcription factors to the RNA polymerase II transcription machinery; HSI2/VAL1 recruits MED13 and HDA6 to suppress a subset of seed maturation genes in cotyledons (Chhun et al., 2016). However, HIGH-LEVEL EXPRESSION OF SUGAR-INDUCIBLE GENE2-LIKE1 (HSL1)/VAL2 but not HSI2/VAL1 can specifically interact with HDA19 through the CW domain; HSL1/VAL2 recruits HDA19 to directly repress the ectopic expression of seed maturation genes in seedlings (Zhou et al., 2013). Mutations in *HDA19* or *HSL1*/*VAL2* lead to the ectopic expression of seed maturation genes associated with the enrichment of gene activation marks (H3ac, H4ac, and H3K4me3) and reduction in levels of gene repression mark H3K27me3 in seedling (Zhou et al., 2013). The absence of *HSL1*/*VAL2* and *HDA19* leads to embryonic lethal (Zhou et al., 2013). Actually, HDA6 and HDA19 may redundantly contribute to the repression of embryonic traits after germination (Tanaka et al., 2008). These findings indicate that the existence of crosstalk among multiple chromatin modifiers for the coordinated control the vegetative-to-embryonic transition in the roots (Figure 1).

PRC1-related factors are also involved in other root developmental events. ALFIN1-like proteins (ALs) can recognize the active mark H3K4me3 through the PHD-domain and interact with the RAWUL domain of PRC1 RING-finger proteins through the PAL domain (Molitor et al., 2014). Mutation in *AL6* causes very short root hairs under phosphate starvation, which actually induces root hair elongation in WT (Chandrika et al., 2013a,b). LHP1 is considered as a Pc equivalent of PRC1 for recognizing the H3K27me3 mark (Gaudin et al., 2001), however, it can also interact with PRC2 subunits MSI1, EMF2, and VRN2 (Derkacheva et al., 2013; Hecker et al., 2015); thus, LHP1 may act as a bridge between PRC1 and PRC2 (Feng and Lu, 2017). The *lhp1* mutant phenotype is closer to PRC1 mutant *emf1* and PRC2 mutants *clf* and *emf2* than to PRC1 mutants *atring1a atring1b* and *atbmi1a atbmi1b*, indicating LHP1 may exert specific function independent of PRC1 RING finger proteins. For instance, unlike PRC1 RING finger proteins repressing embryonic traits in root, LHP1 interacts with SCR to suppress premature middle cortex formation which is derived from extra longitudinal asymmetric cell divisions in the ground tissue (Cui and Benfey, 2009). Furthermore, the premature middle cortex phenotype in *lhp1* or *scr* mutants similar to GA signaling mutants can be suppressed by phytohormone gibberellin (GA) but is enhanced by GA biosynthesis inhibitor paclobutrazol (Paquette and Benfey, 2005; Cui and Benfey, 2009), indicating the interplay between phytohormone signaling and chromatin-based regulation in regulating middle cortex formation.

## Chromatin remodeling in root development

Chromatin-remodeling ATPases in eukaryotes consists of four major subfamilies (SWI/SNF, ISWI, CHD, and INO80/SWR1) and many additional types (Clapier and Cairns, 2009; Han et al., 2015). Thereinto, SWI2/SNF2 subfamily contains four members: BRAHMA (BRM), SPLAYED (SYD), CHR12, and CHR23 (Flaus et al., 2006). In Arabidopsis, CHD3 chromatin remodeler PICKLE (PKL) plays the essential roles in root development. It inhibits the vegetative-to-embryonic revision by repressing the expression of seed maturation genes in post-embryonic roots (Ogas et al., 1999). Mutation of PKL causes a low penetrance of “pkl root” phenotype, which displays a swollen and green primary root with embryonic traits and accumulation of seed storage reserves (Ogas et al., 1999; Aichinger et al., 2009), reminiscent of the phenotype of some PcG mutants, such as *atring1a atring1b, atbmi1a atbmi1b, swn clf*, and *emf2 vrn2*. ChIP experiment showed that PKL can directly target PcG genes *SWN* and *EMF2* in roots; lack of both PKL and its paralog PKR2 can result in reduced H3K27me3 levels and increased expression of a set of PcG target genes in roots, similar to PcG mutant (Aichinger et al., 2009). PKL is involved in most GA-promoted vegetative growth and phase transitions; GA deficiency can dramatically increase the “pkl root” phenotype in *pkl* mutant (Park et al., 2017). PKL is required for root meristem maintenance, and *pkl* mutant displays shorter root and decreased root meristem activity (Aichinger et al., 2011). PcG protein CLF and PKL antagonistically determine root stem cell activity; depletion of CLF leads to increased root meristematic activity (Aichinger et al., 2011). PKL/SSL2 represses auxin-mediated LR initiation through counteracting auxin signaling repressor SLR, which suppresses downstream activators ARF7 and ARF19. Similar to TSA treatment, *pkl* mutation restores LR formation of the gain-of-function allele *slr-1*, as does (Fukaki et al., 2006). In rice, PKL/CHD3 homolog CHR729 regulates multiple developmental events including root growth via the gibberellin pathway; the phenotype of the *chr729* mutant with reduced bioactive GA3 can be partially rescued by exogenous GA3 (Ma et al., 2015). CHR729 can respectively bind to H3K4me2 and H3K27me3 through its chromodomain and PHD domain; disruption of CHR729 gives rise to a global decrease of H3K27me3 and H3K4me3, but not H3K4me2 and H3K4me1 (Hu et al., 2012). These indicate that CHR729 acts as a bifunctional remodeler to read and interpret histone marks (Hu et al., 2012). Another rice CHD protein CROWN ROOTLESS6 (CRL6) is required for the development of shoot-born crown roots via the auxin signaling pathway (Wang et al., 2016).

SWI/SNF remodeler BRM regulates primary root elongation in an ABA-dependent manner, and it can bind to the *ABI5* locus (Han et al., 2012). The *brm* mutant exhibits ABA hypersensitivity and increased drought tolerance, mimicking the ABI5 overexpression phenotype. This finding indicates that BRM mediates the balance between growth and stress responses through the ABA signaling pathway. Additionally, BRM maintains root SCN dependent on the PLETHORA (PLT) pathway by directly targeting the chromatin of several *PIN* genes (P*IN1*–*PIN4*, and *PIN7*) and affecting auxin distribution. *PLT2* overexpression can partially rescue a *brm* SCN defect (Yang et al., 2015). Furthermore, BRM interacts with the SUMO ligase AtMMS21, which can stabilize BRM protein by SUMOylation in roots. *AtMMS21* mutation results in decreased BRM protein level (Zhang et al., 2017). The SWI/SNF complex subunit BAF60 promotes root growth and RAM size through controlling phytohormone cytokinin (CK) production and cell cycle progression via repressing the active histone mark deposition and chromatin loop formation on the key CK biosynthesis genes *IPT3* and *IPT7* and cell cycle inhibitor *KRP7* (Jegu et al., 2015). CHR12/MINUSCULE1 (MINU1) and CHR23/MINU2 are required for root development and RAM maintenance. The *chr12* or *chr23* single mutants display WT-like phenotype, but knockout of both in *chr12 chr23* strong double mutant causes embryonic lethality without root and shoot meristems initiation. In the weak double mutant, dramatically defective RAM including hardly identified QC and irregular columella cell layer results from aberrantly oriented cell division and impaired division rate (Sang et al., 2012). Furthermore, CHR23 can directly bind to the *WOX5* promoter and repress its expression. By contrast, overexpression of *CHR23* reduces root growth due to reduced cell elongation and increases phenotypic variation in accordance with increased expression variability of subsets of environmental stress-related genes among genetically identical individuals (Folta et al., 2014). Therefore, CHR23-mediated chromatin remodeling might provide a buffer system to ensure growth robustness against environmentally-induced phenotypic and transcriptional variation (Folta et al., 2014).

## Conclusions and perspectives

The performance of the root system is dependent on the formation of primary, lateral and adventitious roots. Chromatin modification mainly consists of DNA methylation, histone modification and chromatin remodeling. Although DNA methylation is involved in multiple developmental phases, its role in root development is a mystery and needs systematical investigation. Histone modification and chromatin remodeling has been demonstrated to participate in diverse aspects of root system development, such as root elongation, LR initiation, stress response, cell patterning, stem cell maintenance, cell fate determination, and cell proliferation and differentiation, but most of the related reports only focus on the model plants Arabidopsis and rice. Comparing and elucidating chromatin-based regulatory mechanisms of different root types (like epiphytic root and storage root) in distinct plants will deepen our understanding of root function and phylogenesis. Root developmental progresses is closely associated with stem cell fate transition during RAM differentiation, where the dynamic change and action mechanism of epigenetic marks in specific cell lineage need to be addressed.

Chromatin modifications usually have global yet uneven distribution throughout the plant life cycle, and depletion of each modification type often causes pleiotropic phenotypes in addition to abnormal root development, such as *clf* mutant with curly leaf, early flowering and homeotic conversion of floral organ (Goodrich et al., 1997), and *atring1a atring1b* double mutant with ectopic meristem, twisted blade, late flowering, overproliferated floral organ, and poor fertility (Xu and Shen, 2008; Chen et al., 2010; Chen D. et al., 2016). Such modifications may form different combinations allowing for accurate and fine regulation of developmental processes. (1) Chromatin modifiers with antagonistic functions precisely maintain the homeostasis of chromatin modifications to ensure normal growth and development of plants. (2) Different types of chromatin modifiers may constitute a single multiprotein complex or act together to exert complicated functions. CHD3 family members in animals belong to the NuRD (nucleosome remodeling and deacetylase) complex that participates in transcriptional repression through coupling chromatin remodeling and deacetylation (Bouazoune and Brehm, 2006). Consistently, Arabidopsis CHD3 remodeler PKL and histone acetylation are involved in repressing auxin-mediated LR initiation (Fukaki et al., 2006). (3) Distinct complex variants form dependent on the specific developmental phase. PRC2 complex variants EMF-PRC2, VRN-PRC2, and FIS-PRC2 function in vegetative maintenance, vernalization, and endosperm development, respectively (Bemer and Grossniklaus, 2012). (4) Alteration of work mode occurs in specific developmental context. PRC1-mediated H2Aub1 initiates the repression of seed maturation genes and subsequent recruit PRC2 complex to maintain repression through introducing H3K27me3 marks, different from the classic hierarchical model in animal PcG-mediated repression, where H3K27me3 precedes H2Aub1 (Yang et al., 2013). A chromatin modifier may harbor dual roles. For instance, rice chromatin remodeler OsCHR729 may recognize histone methylation (Hu et al., 2012). These regulatory modes in different developmental events provide essential references for chromatin-based mechanism underlying root development.

To date, only a small number of chromatin modifiers displaying obvious phenotype in single mutants and to a less extent in double mutants have been functionally characterized in the roots, whereas lack of investigations on most chromatin modifiers might be difficult in phenotype analysis due to existence of multiple redundant genes. The newly-developed CRISPR (clustered regularly interspaced short palindromic)/Cas (CRISPR-associated) system provides an extremely powerful and labor-saving tool for targeted gene editing. This tool can be used to target several genes simultaneously for fast and efficient generation of high-order mutants (Cong et al., 2013; Shan et al., 2013). Additionally, the fast-growing multiple omics technologies (transcriptomics, proteomics, and epigenomics) associated with conventional experiments (ChIP, Pull-down, and affinity purification) can be used for high-throughput identification of the target genes and partners of chromatin modifiers to elucidate their functions in root development. Gene transformation is not established in many plants with different root types, thereby limiting the application of the CRISPR/Cas system and production of mutants in these plants. Hence, exploitation of novel inhibitors is preferable to chromatin modifiers for mutant mimicking and human epigenetic disease therapy.

## Author contributions

D-HC and YH wrote the manuscript. CJ collected the raw materials. J-PS provided important suggestions.

### Conflict of interest statement

The authors declare that the research was conducted in the absence of any commercial or financial relationships that could be construed as a potential conflict of interest.
